# Supplementary material for: Spontaneous discovery of novel task solutions in children
Source: PLoS One. 2022 May 31;17(5):e0266253. doi: 10.1371/journal.pone.0266253 (PMC9154107; doi:10.1371/journal.pone.0266253)
Supplement: Attachment — Submitted filename: Response to Reviewers.pdf [file pone.0266253.s001.pdf]

Dr. Nicolas Schuck | Research Group Leader
Max Planck Research Group
Neural and Computational Basis of Learning,
Decision Making and Memory

05.02.2022

We thank both reviewers for their time and insightful comments. We address each point below. Quoted reviewer questions are given in ***bold italics***, and new text in the manuscript is quoted in blue.

Reviewer #1

Dear Dr Laurence,

Thank you for your helpful review and positive comments on our manuscript. We appreciate your input and will address all points below.

Major points

1) The authors mentioned that their lack of evidence of their results in studies 1 and 2 could be an effect of small power (page 24, lines 433-434). Their solution was to combine the data from studies 1 and 2. I think that authors could also conduct a post-hoc achieved power analysis in order to statistically present what is the power that they found when combining both samples.

Thank you for this valid point. We now report on page 12 of our revised manuscript:

"Post-hoc power tests for X^2-tests revealed that with our increased sample size of 86 participants we had a power of .79 to detect effect-size of .3, and .45 power to detect an effect-size of .2 (analyses assuming alpha = .05, two-sided)."

Minor points

1) The plots are presented in shades of blue and red. Thinking that colorblindness affects ~4.5% of the population, it can be interesting to change the color of the plots to shades that are more colorblind-friendly. One example is the Viridis package of R, which presents dark purple (#440154FF) and light yellow (#FDE725FF) as opposing colors. Other examples of colorblind-friendly colors are #999999, #E69F00, #56B4E9, #009E73, #F0E442, #0072B2, #D55E00, #CC79A7. This change can help the interpretability of the paper. I do not think this is a must-do change to the paper, but it would benefit it.

Thank you for raising this important point. All plots in the manuscript have been adapted to zse the purple and yellow color scheme you suggested.

2) Plot A and B of Figure 5 is missing the x-axis title. Please, add the x-axis title in these plots

This issue has been fixed.

Max Planck Institute for Human Development

Directors:
Ute Frevert
Ralph Hertwig
Ulman Lindenberger
Iyad Rahwan

Lentzeallee 94
14195 Berlin
Germany

Tel. +49 30 82406-304
Fax +49 30 8249939
schuck@mpib-berlin.mpg.de
www.mpib-berlin.mpg.de

Reviewer #2

The authors address a worthwhile question — does children's more diffuse attention aid them in the discovery of novel strategies? The authors present a study comparing children's ability to discover novel strategies with that of adults and examined how this related to cognitive control abilities. They found that children and adults did not differ in their discovery of novel strategies despite differences in their overall performance in the task, working memory capacity, and response inhibition.

Thank you for your helpful review and positive comments on our manuscript. We appreciate your input and will address all points below.

1. I question whether it's valid to combine the datasets from experiment 1 and 2. Doubling the stimulus display time is a considerable change and seems like it could have affected the deployment of the instructed color strategy (i.e. in experiment 2, there is not a difference in color use between children and adults after instruction).

We thank the reviewer for this interesting point. We agree in principle, that combining both experiments is not without problems. Yet, the manuscript already presents the results from each experiment individually at great length. Importantly, the combined data is only used to confirm the findings from the Experiments 1 and 2, and no major novel conclusions are derived from the combined analysis.

Nevertheless, we now add a word of caution to the discussion regarding the combined analysis (p. 15):

"Note that any novel insights obtained from combining Experiments 1 and 2 ignore the fact that doubling the stimulus display might have affected the deployment of the color strategy. Hence, new experiments using a constant stimulus display with a larger sample size are warranted "

2. The following points concern the discussion.

a. On page 29, the results are discussed in light of bayesian accounts of developmental change (e.g. Children have weaker priors and hence rely more on the data they encounter). The authors point out that the instructed spatial strategy implements a prior belief that should be shared between children and adults. But, the discussion is very vague regarding why children would be regularizing more towards this prior belief than adults which is what their data from the first experiment suggests.

We thank the reviewer for making us realize that our writing was not clear. With Bayesian accounts, we were referring to the use of the computational model as a general approach to capture parameters such as exploration and exploitation. We now revised the sentence on p. 14 to

"Further investigations are therefore needed that shed light on the factors that facilitate and impede strategy discovery, for instance using Bayesian models to computationally capture parameters of exploitation and exploration directly and utilizing update focused working memory measures such as n-back or AX-CPT tasks. "

b. On page 30, mPFC and its role in novel strategy discovery and its ongoing development at 8-10 is discussed. However, it is not fully addressed what it means that mPFC is still under development yet these behavioral results demonstrate that children are able to discover novel strategies at the same rate as adults.

We thank the reviewer for this important point. We now add to the discussion (p. 15):

"This implies that our sample of children could be characterized by substantial inter-individual differences in mPFC structure. Direct measurement of mPFC structure could therefore be an interesting subject of investigation."

c.How do these results relate to differences in the developmental trajectories of selective attention and response inhibition (Booth, 2003 Neuroimage; Tipper, 1989, Journal of Experimental Child Psychology)? Particularly because of the child age range examined. 8-10 year olds are thought to have relatively mature selective attention but response inhibition continues to develop into adolescence.

We thank the reviewer for bringing up this interesting question. We now added the following to our Discussion:

"While the present study was not designed to specifically examine the question about the factors influencing strategy discovery, the available data thus could hint at two possible explanations. On the one hand, relatively better task and Stroop performance could reflect different mechanisms in adults versus children. Whereas in adults good performance mainly reflects attentional focus that is detrimental to strategy updating, in children relatively good task performance could reflect different factors, such as better encoding of the instructions or better motor control. Interestingly, selective attention is thought to be mature relative to inhibitory control among children in the present study; 8–10 year olds have been found to show only small deficits on selective attention tasks, but large differences on response inhibition tasks, relative to adults (e.g., Booth et al., 2003). This protracted development of response inhibition is congruent with the marked differences in task performance (e.g., on LateGo and No-Go trials) in our current study, and suggests that it may be response inhibition, rather than attentional focus more broadly, that contributes to the ability to spontaneously discover alternative solutions.

On the other hand, given the overall performance difference between children and adults it also seems possible that the opposite-sign relations to strategy switching reflect an inverted U-shaped relationship between attentional mechanisms and strategy switching. Further investigations are therefore needed that shed light on the factors that facilitate and impede strategy discovery, for instance using Bayesian models to computationally capture parameters of exploitation and exploration directly. Furthermore, more differentiated measures of selective attention, response inhibition, and update focused working memory measures such as n-back or AX-CPT tasks should be utilized to tease apart the specific aspects of cognitive control that relate to strategy switching in children and adults, respectively. "

3. The following points concern the Stroop results a. On page 18, there is a footnote explaining that semantic facilitation reflects a failure of cognitive control and reports no age difference in semantic interference. I suggest moving this out of the footnote and providing further explanation for why semantic facilitation reflects a failure of cognitive control. Is this because it demonstrates that they are attending to an irrelevant feature (text)? This feels important for motivating the correlations between main task performance and semantic facilitation later on.

We agree with the reviewer and have moved the footnote into the main text.

The reviewer's interpretation regarding the meaning of the effect is also correct. We have added the necessary clarification to the text on page 9, which now reads as follows:

"Note that because participants were instructed to respond to the ink color, not respond to the written word, the semantic facilitation score reflects processing of the irrelevant stimulus feature (the text of the congruent stimulus), and thus a failure of cognitive control. Surprisingly, we did not find age group differences in semantic interference (neutral - incongruent), which were -30ms and -39ms in children and younger adults, respectively, p = .65"

b. There is a difference between age groups for the semantic facilitation effect but not the semantic interference effect. While not critical to the argument of the paper, I think a quick interpretation of this asymmetry in the discussion would be beneficial in understanding what sorts of inhibitory control show age related differences in this study.

We agree and have now added the following statement to the discussion on page 14:

"[T]he Stroop (semantic facilitation) effect correlated positively with color use among children (r = .35, whereby larger Stroop effects indicate less interference, see Fig. 6D). This hints at a 'beneficial' effect of more cognitive control. Note, however, that we found no age differences in semantic facilitation in our Stroop test, the reasons for which remain unclear and could suggest unreliable measurement."

c. What is "Stroop Costs" in Figure 6a? Is it the same measure referred to as "Stroop effect" in Figure 6D?

The reviewer is correct and the Figure has been adjusted to consistently say Stroop Costs. We have also clarified on page 12:

"Note that all measures in Figure 6 are flipped such that a positive value indicates better performance. For instance, a positive z-score for Stroop costs indicates relatively smaller Stroop costs, while a positive value for RT indicates relatively faster RTs etc"

4. On pages 10-11 lines 190-193, it states that 7 participants' data was lost due to human and technical errors and then 2 participants' data was lost to technical errors. Does this mean 9 participants' data was lost?

The statement in the manuscript was indeed misleading and we thank the reviewer for point this out. We now clarify as follows on page 5:

"In addition to data from participants whose WM scores were lost, Stroop data from two additional participants were lost. Analyses which considered Stroop performance therefore excluded two additional participants, relative to WM analyses."

5. Are the reported correlations on page 26 line 493-495 Pearson? From Figure 6c, it looks like the adult correlation may be driven by the one participant with a very high error rate and very high color use. A spearman rank correlation would be less sensitive to this outlier.

The reviewer is correct that the reported correlations are Pearson, and that the value in the adult group in Fig 6C is inflated by an outlier. Specifically, switching to a spearman correlation changes the adult group value from -.29 to a non-significant value of .22. The value in the children group remains almost unchanged (.30), and significant.

We would like to thank the reviewer for spotting this important issue. Repeating the regression model analysis in a robust regression framework (using the function rlm of the MASS package in R), showed that the effect in question – the interaction effect of Age group with Task performance on strategy change – was not significant anymore. In the case of the Stroop performance (shown in Fig 6D), however, this was still the case (F = 6.334, p-value = 0.01421, using the function 'f.robftest' in R).

In light of these findings, we have now added the following to the main text on page 13:

"Note, however, that only the effect involving Stroop performance (6D), but not the effect of task performance (6C), survived a reanalysis with a robust regression framework. "

6. There are some places where the p-value is reported by not the test statistic (e.g. page 20, line 380 and 382).

We thank the reviewer. The missing test statistic values have now been added.

7.The following are analyses that I don't think are critical for the claims of the paper but I think could clarify some of the results. a. Were there any differences in the distributions of estimated switch points between child and adult color users?

No, the average adult switchpoint (miniblock, only for switching adults) was 6.4, and for children it was 7.3, t(20.6) = -0.86, p = .39. We report this result no on page 12.

b. When child and adult color users (prior to the instructed block) accidentally respond on NoGo Trials, do they tend to respond with the option that corresponds with the color or randomly? Are there age differences in this?

This is an interesting question. We found no evidence of color guided premature responses in NoGo trials at the end of the experiment (blocks 7-8, when color usage was highest) in either children (52.6%, $t(37) = 0.47$, $p = .63$), nor adults (69.7%, $t(18) = 2.0$, $p = .06$). The trend in young adults is notable, but a direct comparison between age groups did also not yield any results, $t(29.2)= -1.5$, $p = 0.14$. Reduced dfs reflect that for some participants no premature responses in NoGo trials were recorded.
